# Correlation between cancer mortality and alcoholic beverage in Japan.

**DOI:** 10.1038/bjc.1979.200

**Published:** 1979-09

**Authors:** S. Kono, M. Ikeda

## Abstract

Geographical correlations between standardized, mortality ratios (SMRs) of cancers and consumption of different types of alcoholic beverages (saké synthetic saké, shochu, beer, wine, and whisky), of cigarettes, and urbanization were examined for all 46 prefectures in Japan. Suggestive correlations were observed between cancer of the oesophagus in males and both shochu and whisky (r = 0.27 and 0.22 respectively), between cancer of the rectum in males and wine (r = 0.45), and between cancer of the prostate and shochu (r = 0.50). These correlations were also confirmed in the partial correlations between SMRs of cancers and consumption of alcoholic beverages, controlling for the two variables urbanization and consumption of cigarettes. Alhtough cancers of other sites were also correlated with certain types of alcoholic beverages, their associations seemed to be secondary to other factors. The validity of higher-order partial correlations and the problems of correlation study are also referred to.


*Br. J. Cancer* (1979) **40**, 449

### CORRELATION BETWEEN CANCER MORTALITY AND ALCOHOLIC BEVERAGE IN JAPAN

S. KONO AND M. IKEDA

*From the Department of Public Health, Faculty of Medicine, Kyushu University, Fukuoka* 812, *Japan*

Received 4 January 1979 Accepted 11 May 1979

**Summary.—Geographical correlations between standardized mortality ratios (SMRs) of cancers and consumption of different types of alcoholic beverages (*saké* synthetic *saké*, *shochu*, beer, wine, and whisky), of cigarettes, and urbanization were examined for all 46 prefectures in Japen. Suggestive correlations were observed between cancer of the oesophagus in males and both *shochu* and whisky ($r$=0·27 and 0·22 respectively), between cancer of the rectum in males and wine ($r$=0·45), and between cancer of the prostate and *shochu* ($r$=0·50). These correlations were also confirmed in the partial correlations between SMRs of cancers and consumption of alcoholic beverages, controlling for the two variables urbanization and consumption of cigarettes. Although cancers of other sites were also correlated with certain types of alcoholic beverages, their associations seemed to be secondary to other factors. The validity of higher-order partial correlations and the problems of correlation study are also referred to.**

ALCOHOLIC BEVERAGES have been reviewed as a risk factor in the development of cancers of the mouth, pharynx, and oesophagus. However, the associations between cancers of other sites and alcohol have not so far been well established (Lowenfels, 1974; Rothman, 1975). On the other hand, it was considered that alcoholic beverages, not alcohol *per se*, may be related to certain cancers (Rothman, 1975). As in other parts of the world there are many kinds of alcoholic beverages in Japan, the consumption of which varies from region to region (Nukada, 1972). *Saké* (fermented product from rice) and *shochu* (distilled spirits made from rice and other grains), which are traditional Japanese liquors, are consumed more in the rural areas, while beer and whisky feature in the urban areas.

There have been several publications on the correlation between cancer and alcohol (Tuyns, 1970; Schoenberg *et al.*, 1971; Breslow & Enstrom, 1974; Enstrom, 1977). However, it is still meaningful to conduct a similar study, using different observational backgrounds, with different patterns and intensities of exposure to aetiological factors (Bjelke, 1974). The present study examined the geographical correlation between cancer mortality of different sites and consumption of different types of alcoholic beverages in Japan, in order to find any clues for distinguishing the causes of cancer, as well as to confirm the reported hypotheses.

### MATERIALS AND METHODS

The materials for the present study consist of data on consumption of alcoholic beverages, of cigarettes, urbanization, and cancer mortality. *Per capita* average annual consumption of alcoholic beverage by type and by prefecture was calculated from the taxed sales for the 3 years from April 1964 to March 1967 (National Tax Administration Agency, 1966, 1967, 1968) and the census population at age of 20 or more in 1965 (Bureau of Statistics, Office of the Prime Minister, 1967). The types of alcoholic beverages analysed are *saké*, synthetic *saké*, *shochu*, beer, wine, and whisky (including brandy). The proportion of

TABLE I.—*Simple correlations between environmental variables*

| | Urban-isation | Cigar-ettes | *Saké* | Syn-thetic *saké* | *Shochu* | Beer | Wine | Whisky |
|---|---|---|---|---|---|---|---|---|
| Cigarettes | 0·83*** | | | | | | | |
| *Saké* | −0·19 | −0·21 | | | | | | |
| Synthetic *saké* | 0·11 | −0·08 | 0·61*** | | | | | |
| *Shochu* | −0·23 | −0·31* | −0·55*** | −0·17 | | | | |
| Beer | 0·94*** | 0·80*** | −0·07 | 0·25 | −0·22 | | | |
| Wine | −0·04 | 0·09 | 0·10 | −0·09 | −0·09 | −0·02 | | |
| Whisky | 0·74*** | 0·65*** | 0·10 | 0·44** | −0·13 | 0·78** | 0·01 | |
| Absolute alcohol | −0·03 | −0·20 | 0·15 | 0·46** | 0·66** | 0·11 | 0·02 | 0·33* |

* $P < 0·05$; ** $P < 0·01$; *** $P < 0·001$.

the amount of each beverage to the total crude amount sold from April 1965 to March 1966 are as follows: *saké*, 33·9%; synthetic *saké*, 2·0%; *shochu*, 6·1%; beer, 54·4%; wine, 1·0%; whisky, 1·8%. The total amount of absolute alcohol was estimated by multiplying the amount of each beverage by the respective approximate concentrations of alcohol (0·40 for whisky, 0·25 for *shochu*, 0·155 for *saké*, 0·15 for synthetic *saké*, 0·12 for wine, and 0·045 for beer).

Since smoking is generally associated with alcohol drinking, *per capita* average annual consumption of cigarettes was also obtained from the taxed sales (Japan Monopoly Corporation, 1965, 1966, 1967) in the same way as that of alcoholic beverage. The degree of urbanization was also included in the present study, because it was considered to affect alcoholic drinking habits. As an index of urbanization, the percentage of the population living in densely inhabited districts to the census population in 1965 (Bureau of Statistics, Office of the Prime Minister, 1967) was used. These 9 variables (urbanization, cigarettes, *saké*, synthetic *saké*, *shochu*, beer, wine, whisky, and total absolute alcohol) are henceforth referred to as the environmental variables.

As for cancer mortality, the standardized mortality ratios (SMRs) for cancer of the oesophagus (8th revised ICD No. 150), stomach (151), colon (153), rectum (154), liver (155, 197·7, 197·8), pancreas (157), lung (162), female breast (174), and prostate (185) during the years 1969–1971 (Segi, 1974) were used.

Correlation coefficients were calculated from the data for all 46 prefectures except Okinawa, which was receded to Japan in 1972.

### RESULTS

### *Preliminary analyses*

As an aid to interpreting the association between cancer mortality and environmental variables, the correlations between male and female SMRs of cancers and those among environmental variables were first analysed.

For all sites of cancer analysed, there were statistically significant correlations between male and female, with wide variation. Correlation coefficients* between male and female were 0·73 for oesophagus, 0·88 for stomach, 0·44 for colon, 0·40 for rectum, 0·80 for liver, 0·60 for pancreas, and 0·33 for lung. A high correlation between male and female may suggest that common aetiological factors are responsible for the cancer in both sexes.

Table I shows the correlation matrix among environmental variables. This table may help to identify some variables which could explain a correlation between SMR of a certain cancer and a particular variable. Some variables were highly correlated with one another. Urbanization, cigarettes, beer and whisky were closely correlated with one another. It is intriguing that *saké* was negatively correlated with *shochu*. Total absolute alcohol was particularly closely correlated with *shochu* and synthetic *saké*.

### *Simple correlations*

Simple correlations between site- and sex-specific SMRs of cancers and environ-

* See the footnote to Table II for statistical significance of correlation coefficients.

TABLE II.—*Simple correlations*† *between SMRs of cancers and environmental variables*

| Site (ICD No.) | Sex | Urban-ization | Cigar-ettes | Type of alcoholic beverage: *Saké* | Synthetic *saké* | *Shochu* | Beer | Wine | Whisky | Absolute alcohol |
|---|---|---|---|---|---|---|---|---|---|---|
| Oesophagus | Male | 0·08 | 0·17 | −0·12 | 0·04 | 0·27 | 0·02 | 0·07 | 0·22 | 0·27 |
| (150) | Female | 0·06 | 0·22 | −0·11 | −0·16 | −0·07 | −0·03 | 0·13 | 0·01 | −0·17 |
| Stomach | Male | 0·05 | 0·15 | 0·41** | 0·18 | −0·51*** | 0·04 | −0·03 | −0·01 | −0·27 |
| (151) | Female | 0·14 | 0·21 | 0·36* | 0·07 | −0·64*** | 0·10 | 0·04 | −0·05 | −0·45** |
| Colon | Male | 0·31* | 0·09 | 0·26 | 0·56*** | −0·07 | 0·40** | 0·04 | 0·34* | 0·33* |
| (153) | Female | 0·24 | 0·18 | 0·47*** | 0·52*** | −0·41** | 0·28 | 0·13 | 0·32* | 0·04 |
| Rectum | Male | 0·03 | 0·11 | 0·11 | 0·13 | −0·07 | 0·03 | 0·45** | 0·12 | 0·07 |
| (154) | Female | 0·03 | 0·00 | 0·30* | 0·35* | −0·32* | 0·07 | −0·09 | 0·15 | −0·09 |
| Liver (155, | Male | −0·17 | −0·25 | −0·12 | −0·22 | 0·33* | −0·14 | 0·18 | −0·26 | 0·22 |
| 197·7, 197·8) | Female | −0·24 | −0·22 | −0·20 | −0·46** | 0·21 | −0·28 | 0·29‡ | −0·40** | −0·06 |
| Pancreas | Male | 0·03 | −0·04 | 0·48*** | 0·46** | −0·04 | 0·10 | −0·01 | 0·26 | 0·42** |
| (157) | Female | −0·03 | −0·10 | 0·57*** | 0·44** | −0·23 | 0·01 | 0·02 | 0·32* | 0·24 |
| Lung | Male | 0·52*** | 0·35* | −0·14 | 0·18 | 0·11 | 0·54*** | −0·04 | 0·42** | 0·26 |
| (162) | Female | 0·43** | 0·29§ | 0·06 | 0·18 | 0·03 | 0·39** | −0·13 | 0·50*** | 0·27 |
| Breast (174) | Female | 0·54*** | 0·55*** | 0·06 | 0·26 | −0·35* | 0·50*** | 0·02 | 0·64*** | −0·11 |
| Prostate (185) | Male | −0·05 | −0·23 | −0·23 | 0·11 | 0·50*** | −0·06 | −0·07 | 0·12 | 0·42** |

† Coefficients of 0·29 and above, 0·38 and above, and 0·47 and above are statistically significant at $P < 0{\cdot}05$, $P < 0{\cdot}01$, and $P < 0{\cdot}001$, respectively.

‡, § Precise values are both 0·285, just below 5% level of significance.

* $P < 0{\cdot}05$, ** $P < 0{\cdot}01$, *** $P < 0{\cdot}001$.

mental variables are summarized in Table II. Since *per capita* consumption of cigarettes and alcoholic beverages is not considered to reflect the amount consumed by women, it is important to examine whether or not the correlations between SMRs and consumption of cigarettes and alcoholic beverages are similar between males and females.

For cancers of the stomach, pancreas, liver and lung, the correlations for males between SMRs and consumption of cigarettes and alcoholic beverages were quite similar to those for females, but with some variation. This was expected to some extent from the correlations between cancer SMRs for males and females. Although the correlations for cancer of the colon were similar between males and females as to some variables, cancer of the colon in males was more highly correlated with beer, while that of females was more highly correlated with *saké* (positively) and with *shochu* (negatively).

The correlations of cancer of the oesophagus in males were quite different from those in females. For males correlations of cancer of the oesophagus with *shochu* and whisky were noteworthy ($r = 0{\cdot}27$ and $0{\cdot}22$ respectively). Similarly for cancer of the rectum, the correlations for males differed from those for females, and a statistically significant correlation was seen only with wine in males ($r = 0{\cdot}45$). Cancer of the breast had a similar pattern of correlation with that of the lung. Cancer of the prostate was closely correlated with *shochu* ($r = 0{\cdot}50$).

The relationships between cancer of the rectum in males and wine, and between cancer of the prostate and *shochu* are plotted in Figs. 1 and 2 respectively. The extreme case visible in Fig. 1 stands for Yamanashi Prefecture. When the correlation coefficient was calculated excluding the extreme value, it turned out to be 0·35, but still remained statistically significant ($P < 0{\cdot}05$). There are also 2 extreme values in Fig. 2. The most ex-

FIG. 1.—Scattergram of correlation between SMR of cancer of the rectum in males 1969–1971 and *per capita* average annual consumption of wine April 1964–March 1967 in 46 prefectures.

FIG. 2.—Scattergram of correlation between SMR of cancer of the prostate 1969–1971 and *per capita* average annual consumption of *shochu* April 1964–March 1967 in 46 prefectures.

TABLE III.—*Partial correlations*† *between SMRs of cancers and alcoholic beverages controlling for urbanity and cigarettes*

| Site (ICD No.) | Sex | *Saké* | Synthetic *saké* | *Shochu* | Beer | Wine | Whisky | Absolute alcohol |
|---|---|---|---|---|---|---|---|---|
| Oesophagus | Male | −0·09 | 0·09 | 0·35* | −0·18 | 0·03 | 0·24 | 0·35* |
| (150) | Female | −0·07 | −0·08 | 0·01 | −0·29 | 0·06 | −0·08 | −0·08 |
| Stomach | Male | 0·46** | 0·26 | −0·49*** | −0·03 | −0·08 | −0·08 | −0·22 |
| (151) | Female | 0·42** | 0·12 | −0·62*** | −0·10 | 0·01 | −0·25 | −0·43** |
| Colon | Male | 0·33* | 0·50*** | −0·07 | 0·38* | 0·15 | 0·20 | 0·28 |
| (153) | Female | 0·54*** | 0·52*** | −0·40** | 0·15 | 0·16 | 0·22 | 0·05 |
| Rectum | Male | 0·14 | 0·19 | −0·03 | 0·01 | 0·43** | 0·14 | 0·13 |
| (154) | Female | 0·31* | 0·36* | −0·34* | 0·12 | −0·08 | 0·20 | −0·11 |
| Liver (155, | Male | −0·18 | −0·29 | 0·27 | 0·06 | 0·23 | −0·19 | 0·16 |
| (197·7, 197·8) | Female | −0·26 | −0·49*** | 0·16 | −0·17 | 0·30* | −0·34* | −0·09 |
| Pancreas | Male | 0·49*** | 0·45** | −0·06 | 0·23 | 0·02 | 0·37* | 0·41** |
| (157) | Female | 0·57*** | 0·42** | −0·28 | 0·12 | 0·05 | 0·52*** | 0·21 |
| Lung | Male | −0·06 | 0·09 | 0·25 | 0·20 | 0·02 | 0·08 | 0·29 |
| (162) | Female | 0·15 | 0·11 | 0·11 | −0·03 | −0·09 | 0·31* | 0·28 |
| Breast (174) | Female | 0·22 | 0·32* | −0·24 | −0·06 | 0·01 | 0·42** | −0·05 |
| Prostate (185) | Male | −0·29 | 0·00 | 0·46** | −0·01 | 0·01 | 0·28 | 0·35* |

† Coefficients of 0·30 and above, 0·39 and above, and 0·48 and above are statistically significant at $*P < 0{\cdot}05$, $**P < 0{\cdot}01$, and $***P < 0{\cdot}001$, respectively.

treme is for Miyazaki Prefecture, and the other for Kagoshima Prefecture. Exclusion of these two values lowered the correlation coefficient to 0·33 ($P < 0{\cdot}05$).

*Partial correlations*

In order to eliminate the effects of urbanization and consumption of cigarettes, partial correlation coefficients were calculated controlling for the two variables. They are summarized in Table III. Compared with simple correlations, partial correlations of cancers of most sites did not change drastically, except for lung and breast. The correlations of cancers of the lung of both sexes and breast with beer decreased to a statistically insignificant level, while those of the lung of female and breast with whisky were still statistically significant. Through this procedure, the correlation between cancer of the oesophagus in males and *shochu* increased to a statistically significant level ($r = 0{\cdot}35$, $P < 0{\cdot}05$).

DISCUSSION

*Validity of higher-order partial correlation*

It may be thought that higher-order partial correlation should have been performed in the present study. We have calculated the partial correlation coefficients between SMRs of cancers and each of 8 environmental variables except total absolute alcohol with other variables constant, but are not convinced of their value.

The multiple correlation coefficients of each type of alcoholic beverage with all other types of beverages were very large, except for wine: *saké* 0·83, synthetic *saké* 0·77, *shochu* 0·70, beer 0·83, wine 0·23, whisky 0·83. To take *saké*, for instance $(0{\cdot}83)^2 \times 100\% = 69\%$ of the geographical variation of *saké* could be explained by 5 other types of beverage. If the partial correlations between SMRs of cancers and *saké* are calculated with the additional 5 variables constant apart from urbanization and cigarettes, it will be the correlation between SMRs of cancers and the small residual portion of the variation of *saké*. Therefore, the higher-order partial correlation of SMRs of cancers with each of the environmental variables were not considered to be useful.

However, it seems worth mentioning the values with regard to wine. The partial correlation coefficients of wine with cancer of the rectum in males and cancer of the liver in females, controlling for all other 7 variables (urbanization, consumption of cigarettes, *saké*, synthetic *saké*, *shochu*, beer, and whisky) were still statistically significant ($r = 0{\cdot}45$, $P < 0{\cdot}01$ and $r = 0{\cdot}33$, $P < 0{\cdot}05$, respectively).

*Site-specific association*

*Cancer of the oesophagus.*—The present study did not reproduce the high correlation between cancer of the oesophagus and alcoholic beverages which has been reported by other correlation studies (Tuyns, 1970; Schoenberg *et al.*, 1971; Breslow & Enstrom, 1974) and by case-control studies (Wynder & Bross, 1961; Martinez, 1969). However, the correlations of cancer of the oesophagus in males with *shochu* and whisky, which were not found for females, indicates that some cases of cancer of the oesophagus in Japan might also be associated with highly concentrated alcoholic beverages.

*Cancers of the rectum and prostate in males.*—The present results with regard to cancer of the rectum did not confirm the findings observed in the correlation studies in the United States (Breslow & Enstrom, 1974; Enstrom, 1977) but the correlation between cancer of the rectum in males and wine was interesting. Although consumption of wine in Japan is very low, it seems to be worth examining in future studies.

The correlation between cancer of the prostate and *shochu* is consistent with the previous studies reporting excess cases of cancer of the prostate among alcoholics (Sundby, 1967; Schmidt & de Lint, 1972; Hakulinen *et al.*, 1974), although case-control studies have not so far claimed alcoholic beverages as a risk factor (Wynder *et al.*, 1971; Steele *et al.*, 1971; Schuman *et al.*, 1977). One of the current hypotheses is that sexual behaviour may be associated with the development of cancer of the prostate (Steele *et al.*, 1971; Schuman *et al.*, 1977). Therefore, the association of the habit of drinking *shochu* not only with cancer of the prostate but also with sexual behaviour should be examined in other studies in Japan.

*Cancers of other sites.*—Positive correlation of cancer of the stomach with *saké* and the negative one with *shochu* are consistent with the correlation study on cancer of the stomach using a different set of data in Japan (Hirayama, 1971).

However, if the correlation of SMR of a certain cancer between males and females is high, and if the patterns of the correlations between its SMR and consumption of cigarettes and alcoholic beverages are similar between males and females, it is reasonable to regard the correlations as secondary to other factors for the reasons mentioned before. When it is also taken into account that there has been no definite excess of death or of cases of cancer of the stomach among cohorts of alcoholics (Sundby, 1967; Schmidt & de Lint, 1972; Hakulinen *et al.*, 1974), and that no increased risk has been observed among daily drinkers of alcohol in the prospective study of Hirayama (1971), the correlations of cancer of the stomach observed here are considered to be secondary to other factors.

For the same reason, the correlations of cancers of the liver and the pancreas with certain types of alcoholic beverages seem unlikely to reflect a causal association between these cancers and any type of alcoholic beverage. However, since cancer of the liver analysed here includes secondary (197·7) and unspecified (197·8) cancer, we hesitate to draw a definite conclusion from the results regarding cancer of the liver. On the other hand, the inference on cancer of the pancreas mentioned above is compatible with the previous observations that there has been no excess of cancer of the pancreas among cohorts of alcoholics (Sundby, 1967; Schmidt & de Lint, 1972; Hakulinen *et al.*, 1974) and that a case-control study has not incriminated alcoholic beverages as a risk factor in the development of cancer of the pancreas (Wynder *et al.*, 1973).

The correlations of cancer of the colon in males with beer and whisky may be consistent with observations in the United States (Breslow & Enstrom, 1974; Enstrom, 1977). However, it would be difficult to admit the causal relation between cancer of the colon and beer drinking, because epidemiological studies on this problem to date are still inconsistent (Enstrom, 1977).

Smoking has been generally known to be causally related to cancer of the lung (Weisburger *et al.*, 1977). The present study also confirmed this, and it was plausible that its correlations with beer and whisky were lowered to a statistically non-significant level in the partial correlations, except the correlation of that of females with whisky. There are several other statistically significant correlations recognized in Tables II and III, especially regarding cancers of the colon and rectum of females and of breast, but we refrain from discussing their significance here, because the discussion would be highly speculative.

*Problems in correlation study*

This type of correlation study is inevitably accompanied by many problems or drawbacks. It goes without saying that taxed sales may not always represent real consumption by prefecture. Additional difficulties in interpreting the results arise from the problems of using populations as sampling units, the long latent interval for most human cancers, and the multiple aetiological factors, which have been intensively discussed in other publications (Breslow & Enstrom, 1974; Enstrom, 1977). Nevertheless, a correlation study is still useful in confirming proposed hypotheses and finding certain clues which will help to elucidate the causes of cancer.

REFERENCES

BJELKE, E. (1974) Epidemiologic studies of cancer of the stomach, colon, rectum; with special emphasis on the role of diet. *Scand. J. Gastroenterol.*, **9**, Suppl. 31, 1.

BRESLOW, N. E. & ENSTROM, J. E. (1974) Geographic correlations between cancer mortality rates and alcohol–tobacco consumption in the United States. *J. Natl Cancer Inst.*, **53**, 631.

BUREAU OF STATISTICS, OFFICE OF THE PRIME MINISTER (1967) 1965 Population Census. Japan.

ENSTROM, J. E. (1977) Colorectal cancer and beer drinking. *Br. J. Cancer*, **35**, 674.

HAKULINEN, T., LEHTIMÄKI, L., LEHTONEN, M. & TEPPO, L. (1974) Cancer mortality among two male cohorts with increased alcohol consumption in Finland. *J. Natl Cancer Inst.*, **52**, 1711.

HIRAYAMA, T. (1971) Epidemiology of stomach cancer. *Gann Monogr.*, **11**, 3.

JAPAN MONOPOLY CORPORATION (1965, 1966, 1967) *The Annual Statistics Reports for the Fiscal Years* 1964, 1965, 1966.

LOWENFELS, A. B. (1974) Alcohol and cancer. *N.Y. State J. Med.*, **74**, 56.

MARTINEZ, I. (1969) Factors associated with cancer of the esophagus, mouth and pharynx in Puerto Rico. *J. Natl Cancer Inst.*, **42**, 1069.

NATIONAL TAX ADMINISTRATION AGENCY, THE MINISTRY OF FINANCE (1966, 1967, 1968) *The Annual Statistics Reports for the Fiscal Years* 1964, 1965, 1966. Japan.

NUKADA, A. (1972) Urbanization and consumption of alcoholic beverages. *J. Hum. Ergol., Tokyo*, **1**, 29.

ROTHMAN, K. J. (1975) Alcohol. In *Persons at High Risk of Cancer*. Ed. J. F. Fraumeni, Jr. New York: Academic Press. p. 139.

SCHMIDT, W. & DE LINT, J. (1972) Causes of death of alcoholics. *J. Stud. Alcohol.*, **33**, 171.

SCHOENBERG, B. S., BAILAR, J. C., III & FRAUMENI, J. F., JR (1971) Certain mortality patterns of esophageal cancer in the United States, 1930–1967. *J. Natl Cancer Inst.*, **46**, 63.

SCHUMAN, L. M., MANDEL, J., BLACKARD, C., BAUER, H., SCARLETT, J. & MCHUGH, R. (1977) Epidemiologic study of prostate cancer: Preliminary report. *Cancer Treat. Rep.*, **61**, 181.

SEGI, M. (1974) *Cancer mortality for selected sites by prefecture in Japan, 1969–1971*. Nagoya: Segi Inst. Cancer Epidemiol.

STEELE, R., LEES, R. E. M., KRAUS, A. S. & RAO, C. (1971) Sexual factors in the epidemiology of cancer of the prostate. *J. Chronic Dis.*, **24**, 29.

SUNDBY, P. (1967) *Alcoholism and Mortality*. Oslo: Universitets-forlaget.

TUYNS, A. J. (1970) Cancer of the oesophagus: Further evidence of the relation to drinking habits in France. *Int. J. Cancer*, **5**, 152.

WEISBURGER, J. H., COHEN, L. A. & WYNDER, E. L. (1977) On the etiology and metabolic epidemiology of the main human cancers. In *Origins of Human Cancer*. Ed. H. H. Hiat, J. D. Watson & J. A. Winsten. Cold Spring Harbor Laboratory. p. 567.

WYNDER, E. L. & BROSS, I. J. (1961) A study of etiological factors in cancer of the esophagus. *Cancer*, **14**, 389.

WYNDER, E. L., MABUCHI, K. & WHITMORE, W. F., JR (1971) Epidemiology of cancer of the prostate. *Cancer*, **28**, 344.

WYNDER, E. L., MABUCHI, K., MARUCHI, N. & FORTNER, J. G. (1973) A case control study of cancer of the pancreas. *Cancer*, **31**, 389.


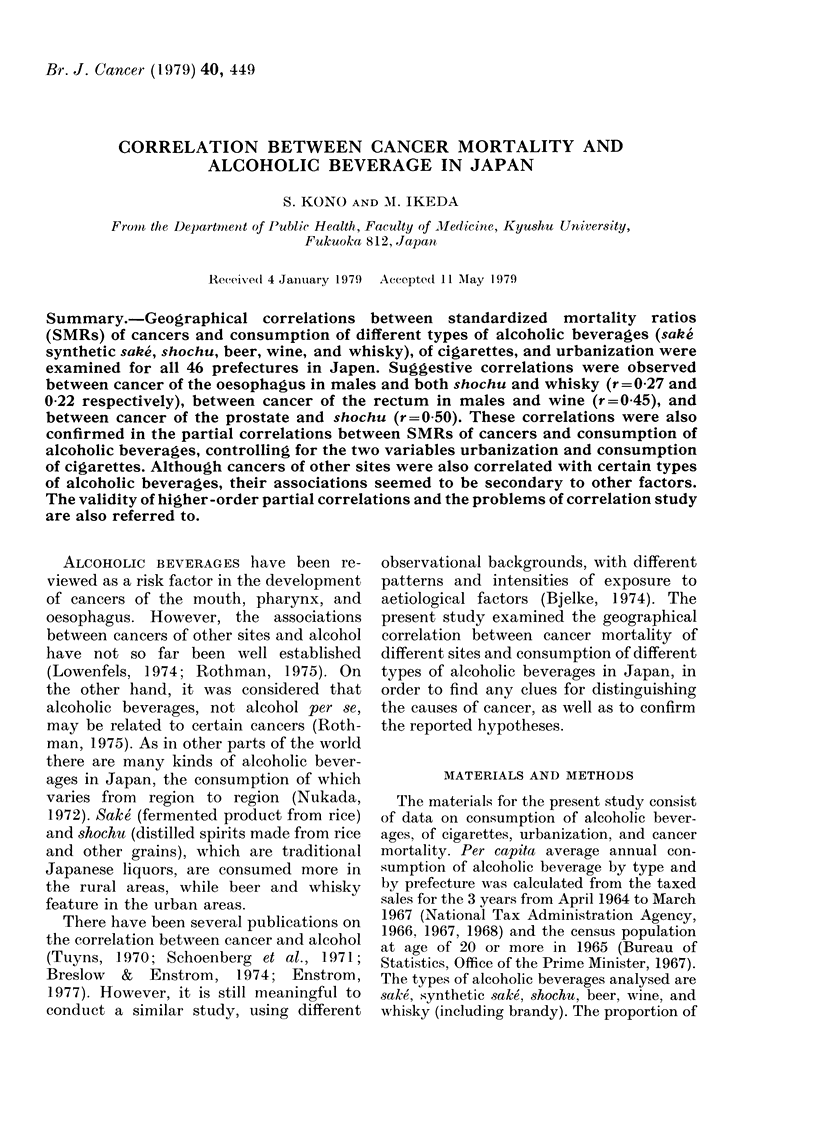

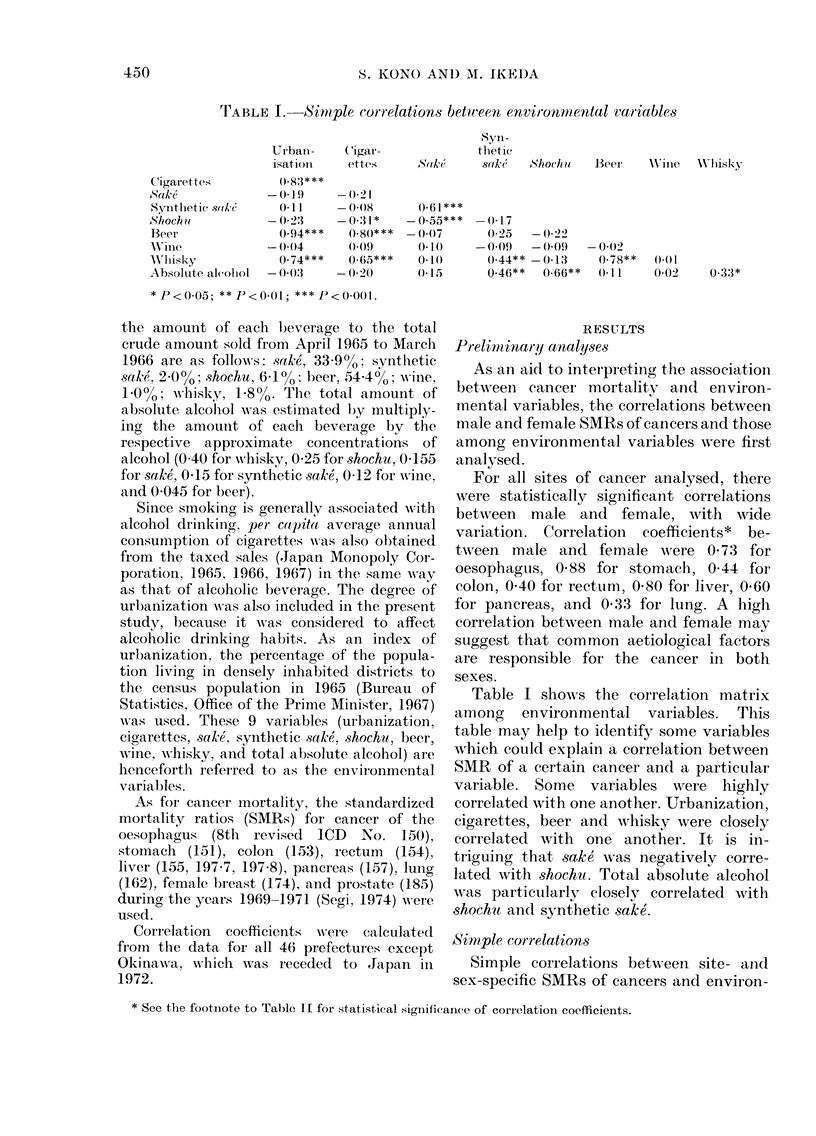

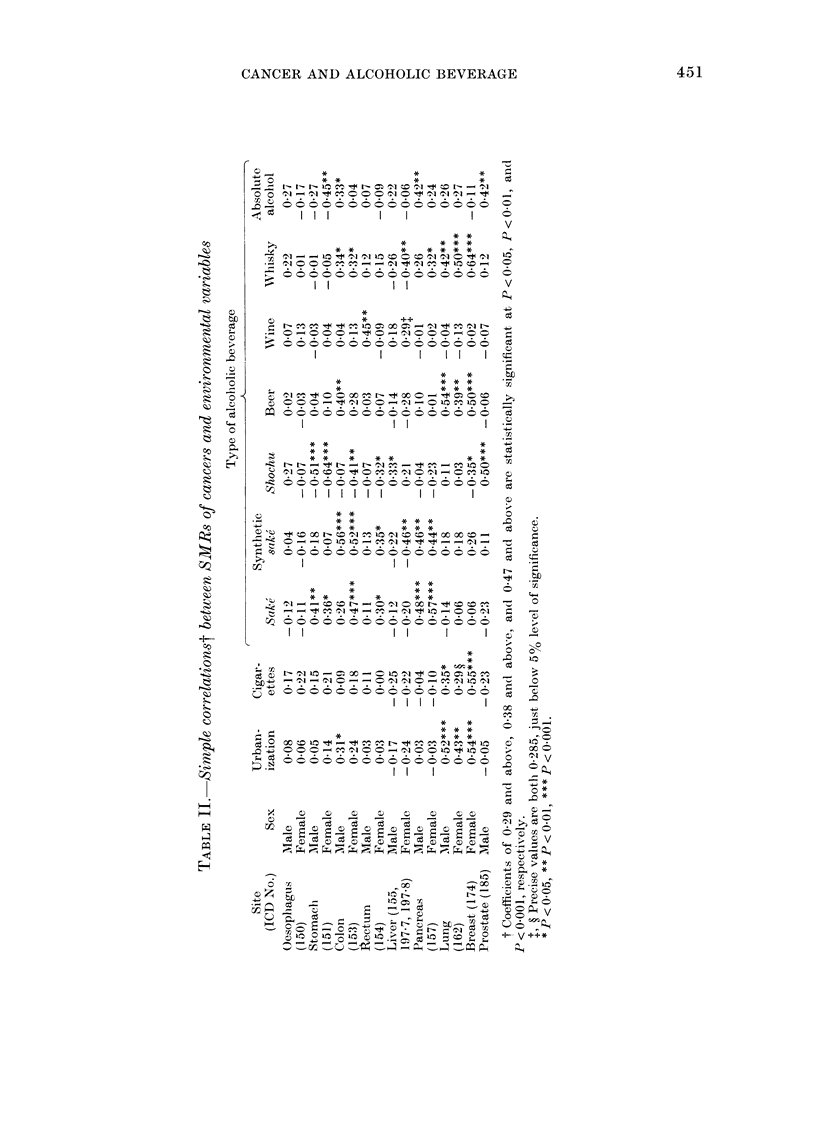

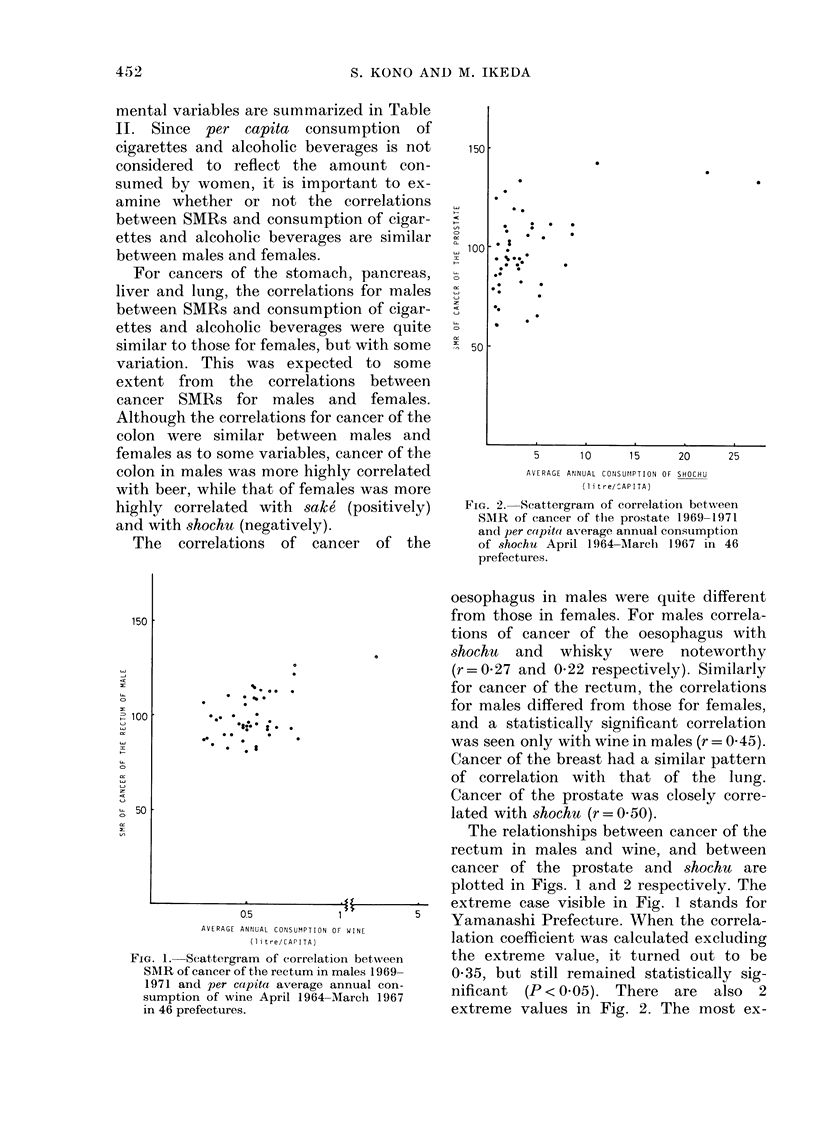

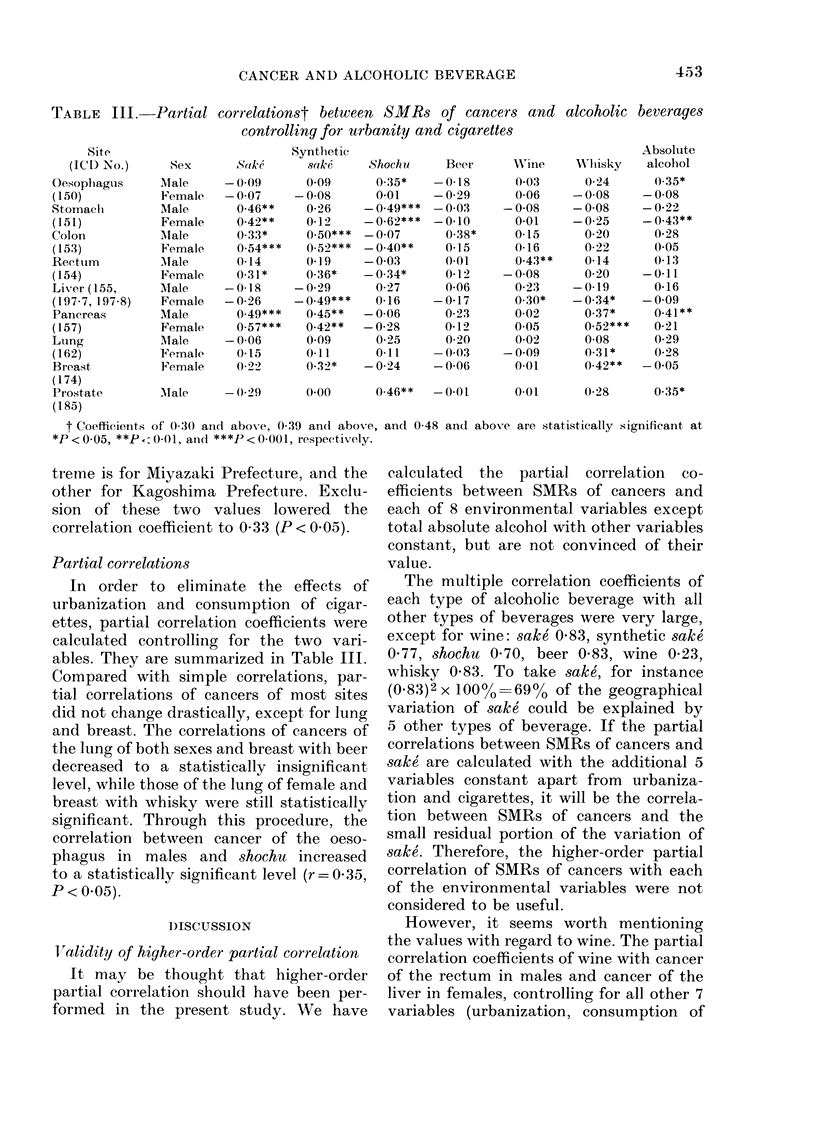

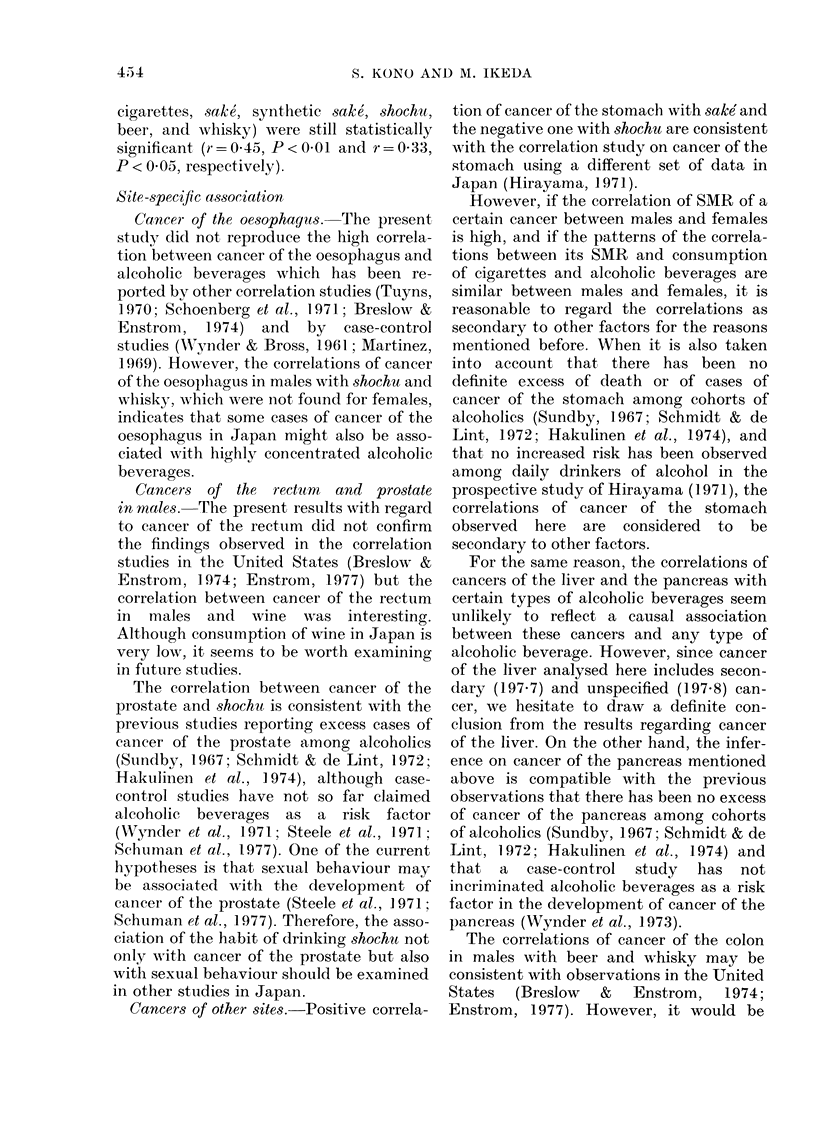

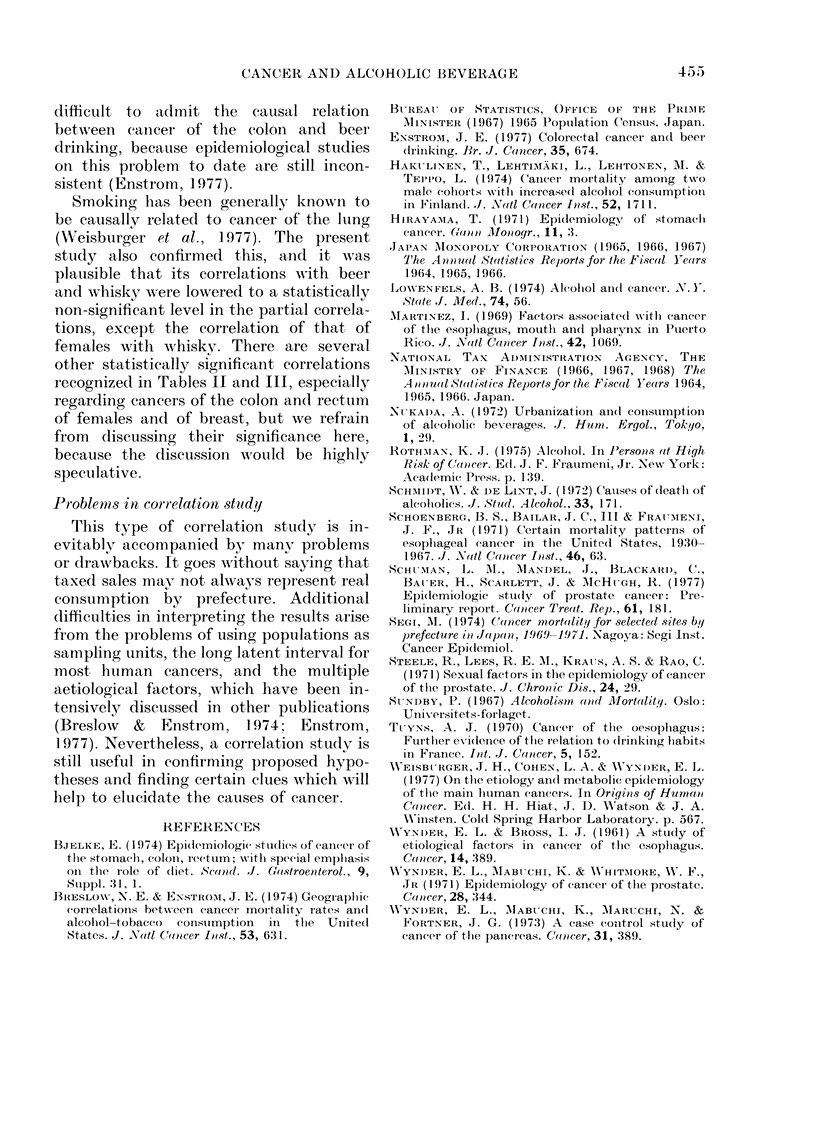


## References

[OCR_00921] Breslow N. E., Enstrom J. E. (1974). Geographic correlations between cancer mortality rates and alcohol-tobacco consumption in the United States.. J Natl Cancer Inst.

[OCR_00931] Enstrom J. E. (1977). Colorectal cancer and beer drinking.. Br J Cancer.

[OCR_00950] Lowenfels A. B. (1974). Alcohol and cancer.. N Y State J Med.

[OCR_01016] WYNDER E. L., BROSS I. J. (1961). A study of etiological factors in cancer of the esophagus.. Cancer.

